# An Interleukin 13 Polymorphism Is Associated with Symptom Severity in Adult Subjects with Ever Asthma

**DOI:** 10.1371/journal.pone.0151292

**Published:** 2016-03-17

**Authors:** Simone Accordini, Lucia Calciano, Cristina Bombieri, Giovanni Malerba, Francesca Belpinati, Anna Rita Lo Presti, Alessandro Baldan, Marcello Ferrari, Luigi Perbellini, Roberto de Marco

**Affiliations:** 1 Unit of Epidemiology and Medical Statistics, Department of Diagnostics and Public Health, University of Verona, Verona, Italy; 2 Section of Biology and Genetics, Department of Neurological, Biomedical and Movement Sciences, University of Verona, Verona, Italy; 3 Unit of Respiratory Medicine, Department of Medicine, University of Verona, Verona, Italy; 4 Unit of Occupational Medicine, Department of Diagnostics and Public Health, University of Verona, Verona, Italy; Chinese Academy of Science, CHINA

## Abstract

Different genes are associated with categorical classifications of asthma severity. However, continuous outcomes should be used to catch the heterogeneity of asthma phenotypes and to increase the power in association studies. Accordingly, the aim of this study was to evaluate the association between single nucleotide polymorphisms (SNPs) in candidate gene regions and continuous measures of asthma severity, in adult patients from the general population. In the Gene Environment Interactions in Respiratory Diseases (GEIRD) study (www.geird.org), 326 subjects (aged 20–64) with ever asthma were identified from the general population in Verona (Italy) between 2007 and 2010. A panel of 236 SNPs tagging 51 candidate gene regions (including one or more genes) was analysed. A symptom and treatment score (STS) and pre-bronchodilator FEV_1_% predicted were used as continuous measures of asthma severity. The association of each SNP with STS and FEV_1_% predicted was tested by fitting quasi-gamma and linear regression models, respectively, with gender, body mass index and smoking habits as potential confounders. The Simes multiple-test procedure was used for controlling the false discovery rate (FDR). SNP rs848 in the IL13 gene region (IL5/RAD50/IL13/IL4) was associated with STS (TG/GG *vs* TT genotype: uncorrected p-value = 0.00006, FDR-corrected p-value = 0.04), whereas rs20541 in the same gene region, in linkage disequilibrium with rs848 (r^2^ = 0.94) in our sample, did not reach the statistical significance after adjusting for multiple testing (TC/CC *vs* TT: uncorrected p-value = 0.0003, FDR-corrected p-value = 0.09). Polymorphisms in other gene regions showed a non-significant moderate association with STS (IL12B, TNS1) or lung function (SERPINE2, GATA3, IL5, NPNT, FAM13A) only. After adjusting for multiple testing and potential confounders, SNP rs848 in the IL13 gene region is significantly associated with a continuous measure of symptom severity in adult subjects with ever asthma.

## Introduction

Asthma is a complex chronic disease that involves the interaction among multiple genetic, environmental and life-style factors. The genetic of asthma has been investigated in many association studies, which have shown that different genes are likely to play a role in disease severity. In fact, polymorphisms in ADRB2 [1], ARG1 and ARG2 [2], CTLA4 [3], IL4 [4], IL4R [5], IL18 [6], TGFB1 [7] and TLR4 [8] genes are associated with asthma severity in several populations. In addition, polymorphisms in ADAM33 are associated with adult-onset asthma and disease severity [9], and this gene and ADAM8 may contribute to the remodelling process that occurs with asthma progression [10]. Moreover, IL6R variation Asp^358^Ala is a potential modifier of lung function in asthma, and it may identify subjects at risk for a more severe form of their disease [11]. In children and young adults, CHI3L1 is associated with asthma-related hospital admissions [12], whereas a polymorphism controlling the expression of ORMDL3 is associated with poor control of the disease in the same age class [13]. In addition, SERPINE1 is related to asthma severity and the response to inhaled corticosteroids [14]. Finally, the expression of IL33 increases in patients with a severe form of the disease [15].

In the 2002 Global INitiative for Asthma (GINA) guidelines [16], the definition of asthma severity was based on a composite categorical classification of lung function, symptom frequency and the daily medication regimen. However, in epidemiological studies, continuous outcomes should be used to catch the heterogeneity of asthma phenotypes that may result from different risk factors. The utilization of continuous measures of disease severity is also justified by the fact that any categorical classification is biologically unsatisfactory for chronic illnesses [17]. In addition, continuous outcomes could increase the power in association studies. Finally, genetic association analyses should be carried out on each dimension of asthma severity (i.e. lung function, symptom frequency and treatment intensity), because different genes can play a role in these different dimensions. In fact, in the French Epidemiological study on the Genetics and Environment of Asthma, new genomic regions were associated with a score of symptom frequency and treatment intensity, but this score had no genetic determinants in common with lung function [18].

The present study is aimed at assessing the association between candidate gene polymorphisms and continuous measures of disease severity in adult subjects with ever asthma, who were identified from the general population in Italy. The analysis was carried out by using the data from the Gene Environment Interactions in Respiratory Diseases (GEIRD) study (www.geird.org) [19].

## Materials and Methods

### Design of the GEIRD study

**Fig 1:**
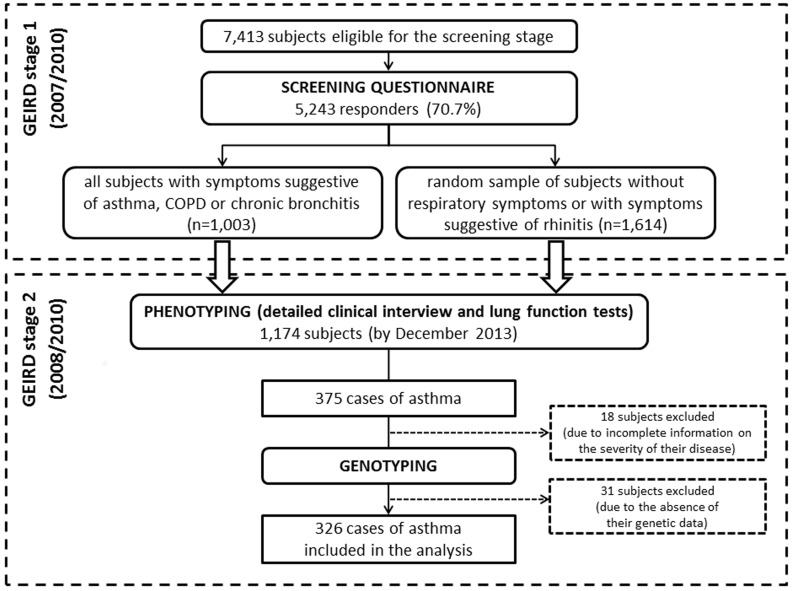
Flow chart of the GEIRD study in the Verona centre and selection of the 326 cases of asthma included in the analysis.

GEIRD is an ongoing, multicentre, (multi)case-control study on respiratory health [19], in which cases and controls are identified through a two-stage screening process (postal questionnaire and clinical examination) in pre-existing cohorts or in new random samples from the general population in Italy. A screening questionnaire (stage 1; 2007/2010) was mailed to 7,413 subjects aged 20–64 (response rate: 70.7%) in the Verona centre. All the responders with symptoms suggestive of asthma, chronic obstructive pulmonary disease (COPD) or chronic bronchitis (n = 1,003), and a random sample of the responders with no respiratory symptoms or with symptoms suggestive of rhinitis (n = 1,614), were invited to undergo a detailed clinical interview and lung function tests for accurate phenotyping, and to provide blood samples for genotyping (stage 2; 2008/2010) (Fig 1). By December 2013, 1,174 of these subjects were examined in the Verona clinic and 1,000 were genotyped. In order to fulfil the purpose of the present study, only the cases of asthma were included in the analysis.

Ethics approval was obtained from the appropriate ethics committee (“*Comitato Etico per la Sperimentazione dell'Azienda Ospedaliera Istituti Ospitalieri di Verona”*). All participants were fully informed about all the aspects of the research project and they gave written informed consent.

### Cases of asthma

In Verona, 375 cases of asthma were identified at the clinical examination. Of these patients, 326 provided genetic data and complete information on the severity of their disease (Fig 1).

A subject was defined as having asthma if he/she fulfilled at least one of the following criteria:

1. having reported ever asthma;
2. having reported asthma-like symptoms [wheezing, nocturnal tightness in the chest, shortness of breath (SoB) following strenuous activities, SoB at rest, SoB at night time] or anti-asthmatic treatment in the past 12 months, and having at least one of the following conditions:
  - airflow hyperresponsiveness (AHR; PD_20_ ≤1 mg);
  - pre-bronchodilator airflow obstruction (AO; FEV_1_/FVC <70% or <Lower Limit of Normal, according to Quanjer [20]) and a positive reversibility test (increase in FEV_1_ >12% and >200 ml with respect to pre-bronchodilator FEV_1_ after 400 mcg of salbutamol);
  - pre- but not post-bronchodilator AO, and post-bronchodilator FEV_1_ ≥80% predicted [20].

### Measures of asthma severity

Two measures of asthma severity were analysed: pre-bronchodilator FEV_1_% predicted, which is as an objective marker of disease severity, and a continuous score of the presence/frequency of respiratory symptoms and of the intensity of anti-asthmatic treatment reported at the clinical interview (symptom and treatment score, STS).

**Fig 2:**
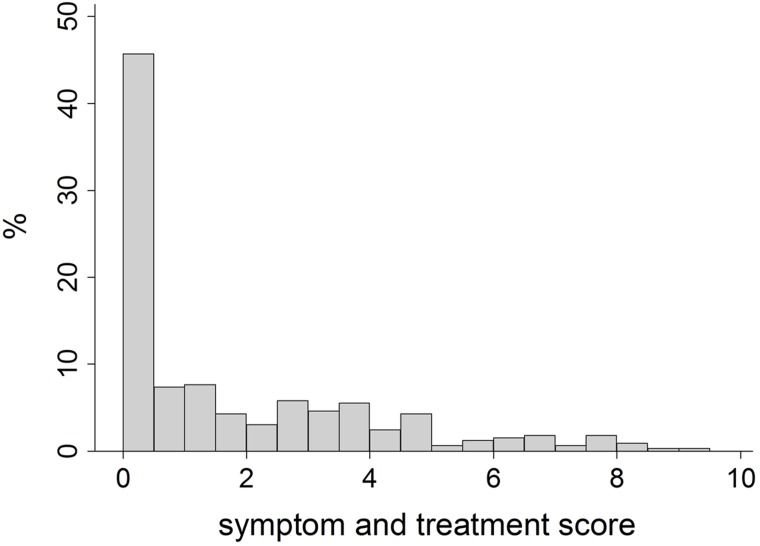
Distribution of the symptom and severity score among the 326 cases of asthma included in the analysis.

STS was obtained as a weighted linear combination of the following variables: (i) frequency of asthma attacks (none, 1–2, 3–11, ≥12) in the past 12 months; (ii) frequency of wheezing (never, sometimes, at least once a week) in the past 12 months; (iii) presence of nocturnal tightness in the chest in the past 12 months; (iv) presence of SoB following strenuous activities in the past 12 months; (v) presence of SoB at rest in the past 12 months; (vi) presence of SoB at night time in the past 12 months; (vii) chronic bronchitis (i.e. cough or phlegm from the chest, usually in winter and on most days for a minimum of three months a year and for at least two successive years); (viii) presence of episodes when symptoms were a lot worse than usual or having reported at least one emergency department visit/hospital admission for respiratory problems in the past 12 months; (ix) intensity of anti-asthmatic treatment [no treatment for asthma, GINA step 1 (only relievers), GINA step 1 (controllers), GINA steps ≥2] in the past 12 months. STS was computed through a multiple factor analysis with the symptoms grouped into a single set of variables [21]. It ranges from 0 (no symptoms/treatment for asthma) to 10 (maximum frequency of symptoms and GINA steps ≥2) and its distribution is approximately an over-dispersed gamma (Fig 2). The concurrent and predictive validity, and the external replication of STS were verified by using the data from the GEIRD study and the European Community Respiratory Health Survey II [22] (data not shown).

### Genetic protocol

In the GEIRD study, a panel of 384 single nucleotide polymorphisms (SNPs), which tag 53 candidate gene regions (including one or more genes), was analysed (S1 Table). The gene regions were chosen on the basis of a previous indication of association with asthma, COPD or rhinitis [from candidate gene or genome-wide association studies (GWAS)], or because of their involvement in biological pathways that are probably related to these respiratory diseases. The selected polymorphisms included both SNPs tagging most of haplotype variability in the CEU population (HapMAp phase II) and candidate SNPs from literature. Genotyping was carried out by means of a customized GoldenGate Genotyping assay.

Two hundred and thirty six SNPs (representative of 51 candidate gene regions) fulfilled the quality control and they had ≤5% of missing values and the minimum genotype frequency ≥5% in our sample. These SNPs were included in the present analysis and were tested according to the additive, dominant and recessive genetic models. The homozygous genotype with the lower allele frequency was considered as the reference.

### Statistical analysis

The association of each SNP with STS and FEV_1_% predicted was evaluated by using quasi-gamma (with log link) [23] and linear regression models, respectively. In order to take the extra variability into account, the quasi-gamma models were fitted through the iterated, reweighted least-squares optimization of the deviance, with the scale parameter set to the Pearson chi-squared statistic divided by the residual degrees of freedom. All the regression models had the genotype (classified according to the additive, dominant or recessive genetic model), gender, body mass index (BMI) and smoking habits as covariates. The strength of the association between each SNP and STS was measured through the ratio of expected scores (RES), whereas the strength of the association with FEV_1_% predicted was measured through the beta regression coefficient, which represents the difference between two genotypes in the expected FEV_1_% predicted. For each outcome, the 708 p-values (obtained after having tested the 236 SNPs according to the three genetic models) were corrected for controlling the false discovery rate (FDR) by using the Simes multiple-test procedure [24].

The statistical analysis was performed by using STATA software, release 13 (StataCorp, College Station, TX), and R software, version 3 (The R Foundation for Statistical Computing, Vienna, Austria).

## Results

### Main characteristics of the asthmatic subjects

The 326 cases of asthma included in the present analysis were on average 42.7 years old (Table 1). Of these individuals, 50.3% were females, 52.8% were ever smokers, and 62.6% had a symptomatic disease (i.e. they had reported the use of anti-asthmatic treatment or at least one asthma attack or at least one asthma-like symptom or the presence of episodes when symptoms were a lot worse than usual or the use of hospital services due to breathing problems in the past 12 months, or coexisting chronic bronchitis). In particular, one out of four patients had used controller medications for asthma in the past 12 months. In addition, 42.6% of the studied individuals had reported an early-onset asthma (<10 years)

**Table 1:** Main characteristics of the asthmatic subjects identified in the GEIRD study, according to their inclusion in the analysis.

		Cases of asthma		
		Included (n = 326)	Not included* (n = 49)	p-value^†^
**Females, %**		50.3	53.1	0.72
**Age (years), mean ± sd**		42.7 ± 9.7	43.9 ± 9.1	0.44
**Smoking habits, %**	Never smoker	47.2	46.9	0.48
	Past smoker	27.0	20.4	
	Current smoker	25.8	32.7	
**BMI, median (interquartile range)**		24.3 (22.0–26.9)	24.7 (21.6–26.7)	0.61
**At least one attack of asthma**^‡^**, %**		17.8	14.6	0.58
**Wheezing**^‡^**, %**	Never	64.1	59.6	0.36
	Sometimes	30.4	29.8	
	At least once a week	5.5	10.6	
**Nocturnal tightness in the chest**^‡^**, %**		17.5	27.7	0.09
**Shortness of breath at rest**^‡^**, %**		8.6	14.9	0.18
**Shortness of breath following strenuous activities**^‡^**, %**		19.9	21.3	0.83
**Shortness of breath at night time**^‡^**, %**		13.2	19.1	0.27
**Chronic bronchitis, %**		13.5	14.9	0.79
**Worsening of symptoms or use of hospital services**^¶^ ^‡^**, %**		14.4	7.0	0.18
**Use of controller medications**^‡^**, %**		24.2	17.9	0.38
**FEV**_**1**_**% predicted, mean ± sd**		102.7 ± 14.5	105.8 ± 14.0	0.42

* Due to the absence of their genetic data or incomplete information on the severity of their disease.

^**†**^ P-values obtained by using two-sample t test, two-sample test on the equality of medians, χ^2^ test or Fisher exact test.

^‡^ In the past 12 months.

^¶^ Presence of episodes when symptoms were a lot worse than usual or having reported at least one emergency department visit/hospital admission for respiratory problems.

The distribution of the demographic and clinical variables, smoking habits and pharmacological treatment for asthma was not significantly different between the studied patients and the 49 cases of asthma that had not been included in the present analysis (due to the absence of their genetic data and/or incomplete information on the severity of their disease) (Table 1).

### SNPs associated with symptom severity

**Fig 3:**
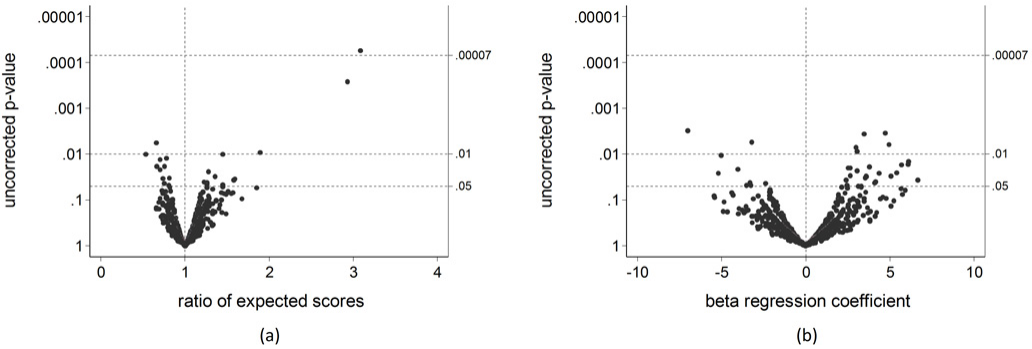
Associations of 236 SNPs in 51 candidate gene regions with the symptom and treatment score (a) and pre-bronchodilator FEV_1_% predicted (b), according to the additive, dominant and recessive genetic models. Data represent the measure of association and the uncorrected p-value for the association of each SNP with the outcome. The FDR-corrected cut-off (0.00007) for statistical significance is reported.

**Fig 4:**
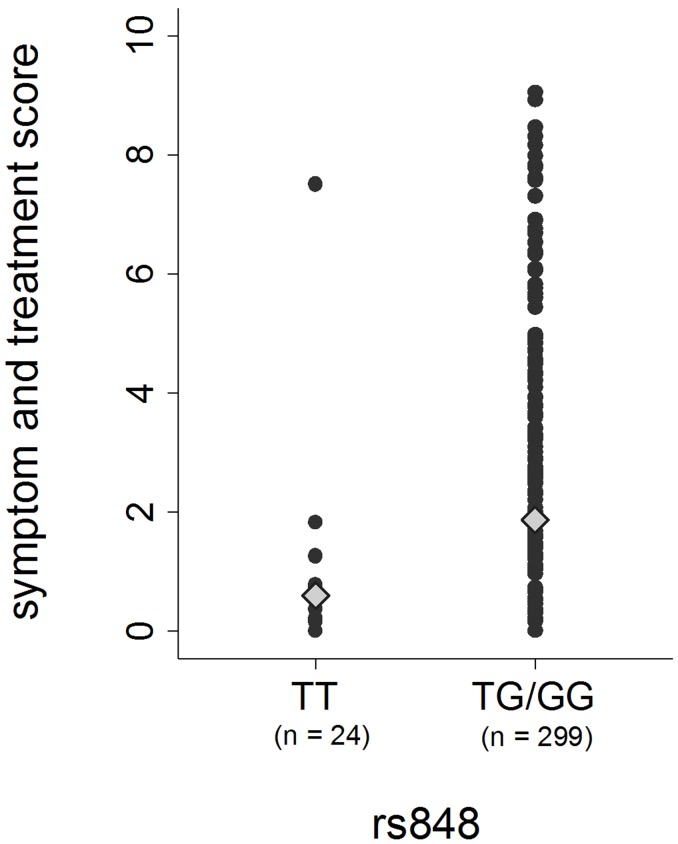
Distribution of the symptom and treatment score among the cases of asthma, according to their genotype in SNP rs848 in the IL13 gene region. The genotype is coded according to the dominant genetic model (the homozygous genotype with the lower allele frequency is the reference). Diamonds represent the expected scores, adjusted for gender, BMI and smoking habits.

Only one SNP (rs848) in the IL13 gene region (IL5/RAD50/IL13/IL4) was significantly associated with symptom frequency and treatment intensity (TG/GG *vs* TT genotype: uncorrected p-value = 0.00006, FDR-corrected p-value = 0.04) (Fig 3). In fact, the subjects with TG or GG genotypes in rs848 had a three-fold increased expected STS as compared to the individuals with TT genotype, the expected score being equal to 1.84 and 0.60 in the two groups of patients, respectively (Table 2, Fig 4). In particular, the TG/GG patients showed a higher risk of reporting wheezing, the use of controller medications for asthma, SoB following strenuous activities and asthma attacks in the past 12 months, as compared to the TT individuals (Table 3).

**Table 2:** Associations with symptom severity.

Gene region	SNP*	Genotype^†^	Genetic model	Number of subjects	Expected STS^‡^ [95%CI]	Ratio of expected scores^‡^ [95%CI]	Uncorrected p-value	FDR-corrected p-value
IL13	rs848	TT	dominant	24	0.60 [0.28 to 0.91]	1.00	-	-
		TG/GG		299	1.84 [1.57 to 2.12]	3.09 [1.78 to 5.34]	0.00006	0.04
IL13	rs20541	TT	dominant	21	0.63 [0.28 to 0.99]	1.00	-	-
		TC/CC		304	1.86 [1.58 to 2.13]	2.93 [1.64 to 5.23]	0.0003	0.09
IL12B	rs2853694	CC/AC	recessive	224	1.99 [1.66 to 2.31]	1.00	-	-
		AA		101	1.31 [0.99 to 1.63]	0.66 [0.49 to 0.89]	0.006	0.99
TNS1	rs929936	TT	dominant	30	0.97 [0.53 to 1.42]	1.00	-	-
		TC/CC		295	1.84 [1.57 to 2.11]	1.89 [1.17 to 3.06]	0.009	0.99

SNP: single nucleotide polymorphism; STS: symptom and treatment score; 95%CI: 95% confidence interval; FDR: false discovery rate.

* Only the SNPs with the uncorrected p-value <0.01 are reported.

^†^ The homozygous genotype with the lower allele frequency is the reference.

^‡^ Adjusted for the effect of gender, BMI and smoking habits.

**Table 3:** Main characteristics of the asthmatic subjects, according to their genotype^*^ in SNP rs848 in the IL13 gene region.

		rs848		
		TT (n = 24)	TG/GG (n = 299)	p-value^†^
**Females, %**		62.5	49.2	0.21
**Age (years), mean ± sd**		40.8 ± 9.8	42.9 ± 9.7	0.32
**Smoking habits, %**	Never smoker	41.7	47.8	0.69
	Past smoker	25.0	26.8	
	Current smoker	33.3	25.4	
**BMI, median (interquartile range)**		24.2 (21.7–25.9)	24.4 (22.0–27.0)	0.56
**At least one attack of asthma**^‡^**, %**		4.2	18.7	0.09
**Wheezing**^‡^**, %**	Never	91.6	62.2	0.005
	Sometimes	4.2	32.1	
	At least once a week	4.2	5.7	
**Nocturnal tightness in the chest**^‡^**, %**		12.5	17.7	0.78
**Shortness of breath at rest**^‡^**, %**		4.2	9.0	0.71
**Shortness of breath following strenuous activities**^**‡**^**, %**		4.2	21.1	0.06
**Shortness of breath at night time**^‡^**, %**		12.5	13.0	1.00
**Chronic bronchitis, %**		12.5	13.7	1.00
**Worsening of symptoms or use of hospital services**^¶^ ^‡^**, %**		16.7	14.4	0.76
**Use of controller medications**^‡^**, %**		4.2	25.4	0.02
**FEV**_**1**_**% predicted, mean ± sd**		101.6 ± 16.0	102.7 ± 14.3	0.70

* Coded according to the dominant genetic model; the homozygous genotype with the lower allele frequency is the reference. Three subjects had no information on SNP rs848.

^**†**^ P-values obtained by using two-sample t test, two-sample test on the equality of medians, χ^2^ test or Fisher exact test.

^‡^ In the past 12 months.

^¶^ Presence of episodes when symptoms were a lot worse than usual or having reported at least one emergency department visit/hospital admission for respiratory problems.

A second SNP (rs20541) in the IL13 gene region, which was in linkage disequilibrium (LD) with rs848 (r^2^ = 0.94) in our sample, was similarly associated with STS, even if this association did not reach the statistical significance after adjusting for multiple testing (TC/CC *vs* TT genotype: uncorrected p-value = 0.0003, FDR-corrected p-value = 0.09) (Table 2). Moreover, a non-significant moderate association with STS was observed for rs2853694 in IL12B (AA *vs* CC/AC) and for rs929936 in TNS1 (TC/CC *vs* TT).

### SNPs associated with lung function

No SNPs were significantly associated with pre-bronchodilator FEV_1_% predicted after adjusting for multiple testing (Fig 3). However, a non-significant moderate association with lung function was observed for the following polymorphisms: rs6721140 in SERPINE2 (AG/AA *vs* GG genotype), rs3802604 in GATA3 (TT *vs* CC/TC), rs2069812 in IL5 (according to the additive genetic model), rs6811135 in NPNT (INTS12/GSTCD/NPNT; according to the additive genetic model) and rs2276936 in FAM13A (GG *vs* TT/TG) gene regions (Table 4). Moreover, rs3802604 (GATA3) and rs2276936 (FAM13A) also showed a non-significant moderate association with lung function according to the additive genetic model, but there was no difference between the reference and heterozygous genotypes in the expected FEV_1_% predicted. These SNPs were not associated with symptom severity.

**Table 4:** Associations with lung function.

Gene region	SNP*	Genotype^†^	Genetic model	Number of subjects	Expected FEV_1_% predicted^‡^ [95%CI]	Beta regression coefficient^‡^ [95%CI]	Uncorrected p-value	FDR-corrected p-value
SERPINE2	rs6721140	GG	dominant	41	109.0 [104.7 to 113.3]	0.0	-	-
		AG/AA		284	102.0 [100.3 to 103.6]	-7.0 [-11.7 to -2.4]	0.003	0.86
GATA3	rs3802604	CC/TC	recessive	185	100.7 [98.7 to 102.8]	0.0	-	-
		TT		139	105.5 [103.1 to 107.9]	4.7 [1.6 to 7.9]	0.004	0.86
IL5	rs2069812	TT	additive	37	97.1 [92.5 to 101.8]^¶^	0.0	-	-
		TC		138	101.9 [99.5 to 104.2]^¶^	3.5 [1.1 to 5.8]^¥^	0.004	0.86
		CC		150	104.7 [102.4 to 107.0]^¶^	7.0 (additive effect)	-	-
NPNT	rs6811135	AA	additive	67	106.7 [103.3 to 110.2]^¶^	0.0	-	-
		AG		169	102.5 [100.4 to 104.7]^¶^	-3.2 [-5.4 to -0.9]^¥^	0.005	0.86
		GG		90	100.2 [97.2 to 103.1]^¶^	-6.4 (additive effect)	-	-
FAM13A	rs2276936	TT/TG	recessive	241	101.4 [99.6 to 103.3]	0.0	-	-
		GG		85	106.4 [103.4 to 109.5]	5.0 [1.4 to 8.5]	0.006	0.86

SNP: single nucleotide polymorphism; 95%CI: 95% confidence interval; FDR: false discovery rate.

* Only the SNPs with the uncorrected p-value <0.01 are reported.

^†^ The homozygous genotype with the lower allele frequency is the reference.

^‡^ Adjusted for the effect of gender, BMI and smoking habits.

^¶^ Estimated after including the genotype as a qualitative variable in the regression model.

^¥^ Estimated after including the genotype as a quantitative variable in the regression model. The beta regression coefficient represents the change in the expected FEV_1_% predicted for 1-unit increase in the genotype variable according to the additive genetic model.

## Discussion

The main results of the present analysis are the following:

- SNP rs848 in the IL13 gene region is significantly associated with a continuous measure of symptom severity in adult subjects with ever asthma, who were identified from the general population in Italy;
- after adjusting for multiple testing, polymorphisms in other gene regions show a non-significant moderate association with symptom severity or lung function only.

### SNPs in the IL13 gene region

Asthma symptoms are related to the presence of activated Th2 cytokine-producing cells (IL4, IL5, IL9 and IL13) in the airway wall [25]. Although each of these cytokines probably contributes to the overall immune response to environmental antigens, IL13 may have a singular role in the regulation of the allergic diathesis [25] and it is a critical mediator of allergic inflammation [26]. In fact, IL13 contributes to many key features of asthma [e.g. mucus production, Immunoglobulin E (IgE) synthesis, bronchial fibrosis and AHR] and consequently it is an attractive target for pharmacological intervention [26,27]. Therefore, a recent Phase II clinical trial has shown that the IL13 blockade with lebrikizumab may improve disease control, even if blocking IL13 is not sufficient to improve lung function [27]. Accordingly, a GWAS has reported that IL13 is not associated with lung function [28].

These findings are supported by the fact that we identified one polymorphism (rs848) in the IL13 gene region, which is significantly associated with a continuous measure of symptom severity but it does not correlate with lung function. In addition, a second SNP (rs20541) in the same gene region, which is in LD with rs848 in our sample, shows a less significant association with the symptom score. This could be due to the relatively small sample size and the strong, but not perfect, LD. However, these two SNPs (or other SNPs in the same LD block) are likely to tag the same gene.

The relationship between rs848/rs20541 and asthma phenotypes has been found in different age classes. In children, the two IL13 polymorphisms are associated with an increased risk of wheezing and both SNPs interact with household carpet use on late-onset asthma [29]. In addition, rs20541 modifies the effect of maternal smoking during pregnancy and household environmental tobacco smoke (ETS) on early-onset persistent wheezing in childhood [30]. The two polymorphisms are also associated with the eosinophil count and serum total IgE in Costa Rican and white (non-Hispanic) children with asthma [31], and rs20541 is related to elevated IgE levels in three Dutch cohorts aged 1–8 [32]. Furthermore, both SNPs are associated with cord blood IgE in Taiwanese full-term new-borns, particularly in males exposed to prenatal ETS [33]. In adults, the role of rs20541 in asthma development has been reported in different studies [34–37]. Moreover, this SNP is associated with AHR in an adult asthmatic Japanese population [38], and it could increase eosinophilic inflammation in the upper and lower airways of patients with aspirin-intolerant asthma [39], which is a severe form of the disease.

To the best of our knowledge, the association between rs20541 and lung function in asthma has been reported with contrasting results [35,38,40–42], whereas no relationship between rs848 and respiratory parameters has been found in African American adults with asthma [40].

### SNPs in other gene regions

We found that rs2853694 in IL12B and rs929936 in TNS1 gene regions show a non-significant moderate association with symptom severity. IL12B may be an important asthma gene [43] and an IL12B promoter polymorphism is associated with the severity of atopic and non-atopic asthma in children [44]. Moreover, a recent GWAS has identified a SNP in TNS1 with suggestive evidence of its association with asthma and coexisting hay fever [45].

In the present analysis, polymorphisms in different gene regions (SERPINE2, GATA3, IL5, NPNT, FAM13A) show a non-significant moderate association with lung function. SERPINE2 is associated with lung function in COPD patients [46], and it has been hypothesized that it is a common genetic determinant of asthma and COPD (i.e. the Dutch hypothesis) [47]. However, our results are in agreement with those from an association study between different SERPINE2 SNPs and asthma-related phenotypes, including lung function, which weakly support this hypothesis [48]. To the best of our knowledge, an association between GATA3, which is an important regulator of T-cell development, and lung function in asthma has been reported in one abstract only [49]. IL5, which is an important cytokine for eosinophil-induced airway inflammation and AHR, is associated with spirometric markers of disease severity in Korean children with atopic asthma [50], whereas the relationship between a locus in INTS12/GSTCD/NPNT and FEV_1_ has been identified in a meta-analysis of GWAS [51]. Finally, FAM13A (a gene associated with COPD [52]) has been shown to predict lung function abnormalities in non-Hispanic white and African American subjects with asthma, together with other lung function genes [53].

### Strengths and weaknesses of our study

The main strength of the present analysis is that our subjects underwent an accurate phenotyping by means of an extended clinical interview and lung function tests [19]. Moreover, the data were collected in patients who had been identified from the general population, rather than from clinically selected groups, which should guarantee that our sample encompasses a wide spectrum of disease severity. In addition, two continuous measures of disease severity were used (STS and pre-bronchodilator FEV_1_% predicted). This choice is justified by the fact that asthma severity is heterogeneous, and respiratory symptoms and lung function may have specific determinants. Accordingly, the use of continuous outcomes, rather than simple categorical definitions, can improve the identification of risk factors by reducing misclassification of the disease status [18]. Finally, a parametric modelling approach was adopted for STS, since it was possible to assume a known theoretical distribution of the score (over-dispersed gamma), with a consequent increase in the power of the association analysis. In fact, if non-parametric tests were used because of the skewness in the score distribution, the association of rs848 with STS would not have reached the statistical significance after the correction for multiple testing (data not shown). Furthermore, parametric models are a powerful tool for controlling the effect of potential confounders, such as gender, BMI and smoking habits.

A few caveats should be taken into account when interpreting our results. At the time of the analysis, the genetic data were collected in a single centre only (Verona), which could limit the generalizability of the results. In addition, both the sample size and the number of SNPs included in the analysis are relatively small. However, the use of continuous measures of asthma severity and the use of appropriate statistical methods made it possible to reduce the weakness of our analysis with regard to the limited sample size.

## Conclusions

After adjusting for multiple testing and potential confounders, SNP rs848 in the IL13 gene region is significantly associated with a continuous measure of symptom severity in adult subjects with ever asthma, who were identified from the general population in Italy. Polymorphisms in other gene regions show a non-significant moderate association with symptom severity or lung function only.

## Supporting Information

S1 Table List of the 384 tag-SNPs in 53 candidate gene regions that were analysed in the GEIRD study. The 236 tag-SNPs included in the association study are reported.
(PDF)

## Abbreviations

95%CI: 95% confidence interval
AHR: Airflow hyperresponsiveness
AO: Airflow obstruction
BMI: Body mass index
CEU: Utah residents with ancestry from Northern and Western Europe
COPD: chronic obstructive pulmonary disease
ETS: environmental tobacco smoke
FDR: False discovery rate
GEIRD: Gene Environment Interactions in Respiratory Diseases
GINA: Global INitiative for Asthma
GWAS: Genome-wide association study
IgE: Immunoglobulin E
IL: Interleukin
LD: Linkage disequilibrium
RES: Ratio of expected scores
SNP: Single nucleotide polymorphism
SoB: Shortness of breath
STS: Symptom and treatment score
