# Supplementary material for: An Interleukin 13 Polymorphism Is Associated with Symptom Severity in Adult Subjects with Ever Asthma
Source: PLoS One. 2016 Mar 17;11(3):e0151292. doi: 10.1371/journal.pone.0151292 (PMC4795623; doi:10.1371/journal.pone.0151292)
Supplement: S1 Table — The 236 tag-SNPs included in the association study are reported. (PDF) [file pone.0151292.s001.pdf]

S1 Table. List of the 384 tag-SNPs in 53 candidate gene regions that were analysed in the GEIRD study. The 236 tag-SNPs included in the association study are reported.

SNP	Gene region	Chr	Chr Pos (GRCh37.p1 assembly)	Included
rs4147567	GSTM1	1	110232524	
rs10857796	GSTM1	1	110235032	
rs2247584	FCER1A	1	159259680	x
rs2427828	FCER1A	1	159273041	x
rs2494265	FCER1A	1	159275274	x
rs2427829	FCER1A	1	159282011	x
rs2275352	CHI3L1	1	203150179	
rs7542294	CHI3L1	1	203151176	
rs946259	CHI3L1	1	203152177	x
rs2071579	CHI3L1	1	203153634	x
rs7515776	CHI3L1	1	203155703	
rs4950928	CHI3L1	1	203155882	
rs10802971	CHML / OPN3	1	241739794	x
rs1053183	CHML / OPN3	1	241758631	
rs3753216	CHML / OPN3	1	241766551	x
rs616593	CHML / OPN3	1	241768617	
rs646355	CHML / OPN3	1	241768630	x
rs3765813	CHML / OPN3	1	241772881	x
rs3753219	CHML / OPN3	1	241775502	x
rs587640	CHML / OPN3	1	241790148	
rs676750	CHML / OPN3	1	241796284	x
rs3819981	CHML / OPN3	1	241800324	x
rs10184597	IL18R1 / IL1RL1 / IL1R1	2	102802255	x
rs3771199	IL18R1 / IL1RL1 / IL1R1	2	102806613	x
rs17637748	IL18R1 / IL1RL1 / IL1R1	2	102841715	x
rs11692230	IL18R1 / IL1RL1 / IL1R1	2	102855065	x
rs17689452	IL18R1 / IL1RL1 / IL1R1	2	102864681	x
rs12475055	IL18R1 / IL1RL1 / IL1R1	2	102878891	
rs12996772	IL18R1 / IL1RL1 / IL1R1	2	102947201	x
rs12712142	IL18R1 / IL1RL1 / IL1R1	2	102960584	
rs10439410	IL18R1 / IL1RL1 / IL1R1	2	102990788	x
rs6758936	IL18R1 / IL1RL1 / IL1R1	2	102991369	x
rs1427680	CTLA4	2	204729795	x
rs11571315	CTLA4	2	204730901	x
rs4553808	CTLA4	2	204731005	
rs16840252	CTLA4	2	204731519	
rs207908	XRCC5	2	217015947	x
rs3821107	XRCC5	2	217031328	x
rs3770507	XRCC5	2	217031678	x
rs207939	XRCC5	2	217042498	x
rs207945	XRCC5	2	217045939	x

rs2161969	TNS1	2	218680570	x
rs4674217	TNS1	2	218681575	x
rs12466972	TNS1	2	218683907	x
rs6729330	TNS1	2	218694296	x
rs16858320	TNS1	2	218699505	
rs929936	TNS1	2	218709145	x
rs987338	TNS1	2	218711738	x
rs3791978	TNS1	2	218714686	x
rs3791961	TNS1	2	218721834	x
rs2042542	TNS1	2	218736292	x
rs3791947	TNS1	2	218739125	x
rs4674220	TNS1	2	218742002	x
rs6729299	TNS1	2	218750154	
rs3828282	TNS1	2	218779144	x
rs13022785	TNS1	2	218782820	x
rs4672859	TNS1	2	218789397	
rs3791906	TNS1	2	218803329	
rs7597833	SERPINE2	2	224842150	x
rs6721140	SERPINE2	2	224858643	x
rs3795877	SERPINE2	2	224866177	x
rs13411332	SERPINE2	2	224887191	x
rs920251	SERPINE2	2	224892945	x
rs164640	TLR9	3	52247314	
rs352139	TLR9	3	52258372	x
rs352143	TLR9	3	52264907	
rs353547	TLR9	3	52268866	x
rs2284659	SOD3	4	24794797	x
rs699473	SOD3	4	24796803	x
rs2536512	SOD3	4	24801315	
rs1799895	SOD3	4	24801834	
rs17552548	SOD3	4	24804722	
rs11944668	FAM13A	4	89669852	x
rs2276936	FAM13A	4	89726283	x
rs1870339	FAM13A	4	89727656	
rs1379934	FAM13A	4	89755842	
rs7674313	FAM13A	4	89777061	
rs6830970	FAM13A	4	89777081	
rs10049947	FAM13A	4	89790593	x
rs1458562	FAM13A	4	89853598	x
rs10008568	FAM13A	4	89854192	x
rs987314	FAM13A	4	89862169	x
rs2904259	FAM13A	4	89885714	
rs1921681	FAM13A	4	89904124	x
rs6844655	FAM13A	4	89915359	x
rs1795739	FAM13A	4	89930313	x
rs1398942	FAM13A	4	89930392	x
rs1795721	FAM13A	4	89943473	
rs11726569	INTS12 / GSTCD / NPNT	4	106606608	
rs2112047	INTS12 / GSTCD / NPNT	4	106698700	
rs2553434	INTS12 / GSTCD / NPNT	4	106798457	
rs2544425	INTS12 / GSTCD / NPNT	4	106801279	

rs6811135	INTS12 / GSTCD / NPNT	4	106830232	x
rs7677312	INTS12 / GSTCD / NPNT	4	106851856	
rs4635819	INTS12 / GSTCD / NPNT	4	106858898	
rs6817700	INTS12 / GSTCD / NPNT	4	106891531	x
rs1489758	HHIP	4	145574239	x
rs6537309	HHIP	4	145605075	x
rs17721701	HHIP	4	145618719	x
rs2087826	HHIP	4	145639318	x
rs3763084	PDE4D	5	58272116	
rs7737685	PDE4D	5	58289046	
rs4700316	PDE4D	5	58294691	
rs2968005	PDE4D	5	58318052	x
rs1948651	PDE4D	5	58333340	x
rs16889129	PDE4D	5	58349882	
rs10061553	PDE4D	5	58352210	x
rs6867053	PDE4D	5	58365545	x
rs929820	PDE4D	5	58393332	x
rs6898374	PDE4D	5	58396902	x
rs10071163	PDE4D	5	58409273	x
rs13186012	PDE4D	5	58427198	
rs13176475	PDE4D	5	58428799	x
rs2409629	PDE4D	5	58478692	x
rs8180396	PDE4D	5	58481760	x
rs9292197	PDE4D	5	58501622	x
rs11747104	PDE4D	5	58505337	
rs153982	PDE4D	5	58520536	
rs27548	PDE4D	5	58527071	x
rs28054	PDE4D	5	58531652	x
rs4699939	PDE4D	5	58552877	x
rs26709	PDE4D	5	58568473	x
rs10491352	PDE4D	5	58573936	
rs27184	PDE4D	5	58575323	x
rs17780175	PDE4D	5	58589786	x
rs17780860	PDE4D	5	58623098	
rs40122	PDE4D	5	58628717	x
rs1823066	PDE4D	5	58675953	x
rs433565	PDE4D	5	58681898	x
rs6874460	PDE4D	5	58701418	
rs258129	PDE4D	5	58711707	x
rs2547918	PDE4D	5	58717094	
rs17795596	PDE4D	5	58722921	
rs16889903	PDE4D	5	58782668	x
rs16889907	PDE4D	5	58785305	
rs1605275	PDE4D	5	58815363	
rs37574	PDE4D	5	58832869	x
rs17797781	PDE4D	5	58834124	x
rs40216	PDE4D	5	58836950	x
rs294498	PDE4D	5	58876950	
rs2069812	IL5 / RAD50 / IL13 / IL4	5	131879916	x
rs6871536	IL5 / RAD50 / IL13 / IL4	5	131969874	
rs2240032	IL5 / RAD50 / IL13 / IL4	5	131977127	

rs1800925	IL5 / RAD50 / IL13 / IL4	5	131992809	
rs20541	IL5 / RAD50 / IL13 / IL4	5	131995964	x
rs848	IL5 / RAD50 / IL13 / IL4	5	131996500	x
rs2243250	IL5 / RAD50 / IL13 / IL4	5	132009154	
rs2070874	IL5 / RAD50 / IL13 / IL4	5	132009710	
rs2243282	IL5 / RAD50 / IL13 / IL4	5	132016554	
rs778584	CD14	5	140005212	x
rs2563298	CD14	5	140011315	x
rs2569190	CD14	5	140012916	x
rs2569193	CD14	5	140015495	x
rs4357026	SPINK5	5	147457939	x
rs2303064	SPINK5	5	147480080	
rs2303065	SPINK5	5	147480112	
rs3777143	SPINK5	5	147487479	x
rs1862439	SPINK5	5	147494931	x
rs2052532	SPINK5	5	147496307	x
rs11743440	SPINK5	5	147499080	x
rs7733401	HTR4	5	147833281	x
rs6887366	HTR4	5	147851270	
rs4264931	HTR4	5	147874556	x
rs17720733	HTR4	5	147950478	
rs867522	HTR4	5	147966246	
rs2910098	HTR4	5	147978253	x
rs2964276	HTR4	5	147978978	x
rs1042713	ADRB2	5	148206440	
rs1042714	ADRB2	5	148206473	x
rs1042717	ADRB2	5	148206646	
rs1042719	ADRB2	5	148207447	x
rs953569	HAVCR1	5	156477400	x
rs1553318	HAVCR1	5	156479323	x
rs6420075	HAVCR1	5	156486120	
rs2853694	IL12B	5	158749088	x
rs730691	IL12B	5	158756227	x
rs1736927	HLAG	6	29796115	x
rs1063320	HLAG	6	29798749	x
rs1610696	HLAG	6	29798803	x
rs2523793	HLAG	6	29802550	x
rs2735014	HLAG	6	29805809	x
rs909253	TNFA / LTA	6	31540313	x
rs2229094	TNFA / LTA	6	31540556	
rs1041981	TNFA / LTA	6	31540784	
rs1800630	TNFA / LTA	6	31542476	
rs1800629	TNFA / LTA	6	31543031	
rs3093662	TNFA / LTA	6	31544189	
rs3093665	TNFA / LTA	6	31545391	
rs2269425	AGER / PPT2	6	32123639	
rs10947233	AGER / PPT2	6	32124424	
rs3134603	AGER / PPT2	6	32126002	
rs3134950	AGER / PPT2	6	32127477	x
rs2269423	AGER / PPT2	6	32145707	x
rs3134945	AGER / PPT2	6	32146492	

rs9469089	AGER / PPT2	6	32146657	
rs3132965	AGER / PPT2	6	32146997	
rs3130349	AGER / PPT2	6	32147696	
rs3134943	AGER / PPT2	6	32147761	
rs1035798	AGER / PPT2	6	32151222	x
rs2070600	AGER / PPT2	6	32151443	
rs3131300	AGER / PPT2	6	32151934	
rs1800684	AGER / PPT2	6	32151994	
rs3129876	HLADRB1 / HLADRA	6	32408012	x
rs3129881	HLADRB1 / HLADRA	6	32409484	x
rs9268659	HLADRB1 / HLADRA	6	32410941	x
rs2213585	HLADRB1 / HLADRA	6	32413150	x
rs3129889	HLADRB1 / HLADRA	6	32413545	
rs9268852	HLADRB1 / HLADRA	6	32429594	
rs9268877	HLADRB1 / HLADRA	6	32431147	x
rs5020946	HLADRB1 / HLADRA	6	32450089	x
rs9269794	HLADRB1 / HLADRA	6	32549249	
rs701831	HLADRB1 / HLADRA	6	32549395	
rs2157337	HLADRB1 / HLADRA	6	32609122	
rs9385992	GPR126	6	142633627	
rs1891308	GPR126	6	142648235	x
rs9389986	GPR126	6	142661114	x
rs11155242	GPR126	6	142691549	x
rs17071756	GPR126	6	142715195	x
rs898070	NPSR1	7	34698865	x
rs1419835	NPSR1	7	34723920	
rs10241507	NPSR1	7	34743478	x
rs1419779	NPSR1	7	34813308	x
rs6462579	NPSR1	7	34866287	
rs764269	NPSR1	7	34875729	
rs10246825	NPSR1	7	34877034	x
rs10258734	NPSR1	7	34896123	
rs1800783	NOS3	7	150689397	x
rs1800779	NOS3	7	150689943	x
rs1799983	NOS3	7	150696111	
rs3918227	NOS3	7	150700946	
rs3918188	NOS3	7	150702781	x
rs1808593	NOS3	7	150708302	
rs7830	NOS3	7	150709571	x
rs892248	ANGPT1	8	108422946	x
rs1342326	IL33	9	6190076	
rs3939286	IL33	9	6210099	x
rs928413	IL33	9	6213387	x
rs10975498	IL33	9	6226688	
rs2006682	IL33	9	6227045	x
rs10815388	IL33	9	6232242	x
rs7019575	IL33	9	6243935	x
rs10975516	IL33	9	6247693	x
rs1330383	IL33	9	6251507	x
rs12000491	IL33	9	6257367	
rs1927914	TLR4	9	120464725	x

rs2770146	TLR4	9	120473338	x
rs5030728	TLR4	9	120474282	x
rs11536897	TLR4	9	120480010	
rs485411	GATA3	10	8093185	
rs3802604	GATA3	10	8102272	x
rs376397	GATA3	10	8103298	
rs3802600	GATA3	10	8108812	
rs10905284	GATA3	10	8115362	x
rs573122	MS4A2	11	59844919	x
rs547110	MS4A2	11	59846718	
rs1441586	MS4A2	11	59856028	
rs2847667	MS4A2	11	59859609	x
rs2583471	MS4A2	11	59861814	
rs569108	MS4A2	11	59863104	
rs174547	FADS1 / FADS2	11	61570783	x
rs174548	FADS1 / FADS2	11	61571348	x
rs968567	FADS1 / FADS2	11	61595564	
rs174579	FADS1 / FADS2	11	61605613	x
rs6591251	GSTP1	11	67344689	x
rs762803	GSTP1	11	67352256	x
rs1695	GSTP1	11	67352689	
rs749174	GSTP1	11	67353253	x
rs1138272	GSTP1	11	67353579	
rs476391	MMP12	11	102735030	
rs651159	MMP12	11	102736419	
rs632009	MMP12	11	102738499	x
rs11225442	MMP12	11	102739319	
rs7123600	MMP12	11	102741968	x
rs2276109	MMP12	11	102745791	
rs5744280	IL18	11	112016514	x
rs549908	IL18	11	112020916	x
rs5744256	IL18	11	112022848	
rs360722	IL18	11	112026703	
rs795467	IL18	11	112031080	x
rs2043055	IL18	11	112031624	x
rs360718	IL18	11	112034739	x
rs757343	VDR	12	48239675	
rs2248098	VDR	12	48253356	x
rs3819545	VDR	12	48265006	
rs10875693	VDR	12	48269650	x
rs11168275	VDR	12	48272275	x
rs2254210	VDR	12	48273714	x
rs2238136	VDR	12	48277713	
rs4760648	VDR	12	48280665	x
rs11168287	VDR	12	48285414	x
rs4334089	VDR	12	48286015	x
rs4760658	VDR	12	48296486	x
rs3024974	STAT6	12	57492745	
rs841718	STAT6	12	57492996	
rs3024957	STAT6	12	57498035	
rs324011	STAT6	12	57502182	x

rs167769	STAT6	12	57503775	x
rs12298170	STAT6	12	57515363	x
rs17119494	STAT6	12	57516933	
rs1732886	IRAK3	12	66583762	x
rs1168771	IRAK3	12	66588836	x
rs1168774	IRAK3	12	66591487	x
rs1168757	IRAK3	12	66594116	
rs1732877	IRAK3	12	66599144	x
rs1152888	IRAK3	12	66605228	
rs2111059	INFG	12	68541671	
rs11177072	INFG	12	68542541	
rs11177073	INFG	12	68542895	
rs2069718	INFG	12	68550162	x
rs2069716	INFG	12	68550815	
rs1861493	INFG	12	68551196	
rs9658490	NOS1	12	117670298	
rs12830203	NOS1	12	117688499	x
rs11068428	NOS1	12	117693817	x
rs4766842	NOS1	12	117720130	x
rs733334	NOS1	12	117732689	x
rs7295972	NOS1	12	117747368	x
rs1483757	NOS1	12	117761540	x
rs545654	NOS1	12	117777049	x
rs11635145	SMAD3	15	67370121	x
rs7181878	SMAD3	15	67389161	x
rs9302242	SMAD3	15	67389412	x
rs4776890	SMAD3	15	67393045	x
rs7163381	SMAD3	15	67414055	
rs11636161	SMAD3	15	67418104	x
rs2118610	SMAD3	15	67428334	x
rs745103	SMAD3	15	67435075	x
rs2289263	SMAD3	15	67439207	x
rs10152544	SMAD3	15	67444747	x
rs744910	SMAD3	15	67446785	x
rs7183244	SMAD3	15	67461311	x
rs12708492	SMAD3	15	67467541	x
rs3784681	SMAD3	15	67472185	x
rs3743343	SMAD3	15	67486775	x
rs11639224	IREB2	15	78753371	x
rs1964678	IREB2	15	78754000	x
rs8043227	IREB2	15	78768871	x
rs647041	CHRNA5 / CHRNA3 / CHRNAB4	15	78880481	x
rs578776	CHRNA5 / CHRNA3 / CHRNAB4	15	78888400	x
rs2869546	CHRNA5 / CHRNA3 / CHRNAB4	15	78907345	x
rs8042059	CHRNA5 / CHRNA3 / CHRNAB4	15	78907859	x
rs1878399	CHRNA5 / CHRNA3 / CHRNAB4	15	78912003	x
rs11636605	CHRNA5 / CHRNA3 / CHRNAB4	15	78928878	x
rs2297516	NOS2	17	26095730	x
rs11080344	NOS2	17	26104511	x
rs4795067	NOS2	17	26106675	x
rs944725	NOS2	17	26109571	x

rs4795400	ORMDL3 / GSDMB / GSDMA	17	38067020	x
rs12603332	ORMDL3 / GSDMB / GSDMA	17	38082807	x
rs3744246	ORMDL3 / GSDMB / GSDMA	17	38084350	x
rs7207600	ORMDL3 / GSDMB / GSDMA	17	38091660	x
rs8065126	ORMDL3 / GSDMB / GSDMA	17	38099035	x
rs7212938	ORMDL3 / GSDMB / GSDMA	17	38122680	
rs921651	ORMDL3 / GSDMB / GSDMA	17	38133922	
rs2241718	TGFB1	19	41829606	
rs4803455	TGFB1	19	41851509	x
rs1800470	TGFB1	19	41858921	
rs1800469	TGFB1	19	41860296	x
rs2317130	TGFB1	19	41861674	x
rs2241713	TGFB1	19	41869468	x
rs677044	ADAM33	20	3649431	
rs2280091	ADAM33	20	3650234	
rs2853209	ADAM33	20	3651472	
rs3918395	ADAM33	20	3653149	
rs511898	ADAM33	20	3655085	x
rs3918392	ADAM33	20	3655219	
rs2853210	ADAM33	20	3658211	x
rs487377	ADAM33	20	3658931	
rs570269	ADAM33	20	3659647	x
rs554743	ADAM33	20	3662142	
rs3918249	MMP9	20	44638136	
rs2274755	MMP9	20	44639692	
rs17576	MMP9	20	44640225	x
rs2236416	MMP9	20	44640575	
rs9607267	HMOX1	22	35781207	
rs11912889	HMOX1	22	35783617	
rs84460	IL2RB	22	37525731	x
rs228945	IL2RB	22	37525880	
rs2072861	IL2RB	22	37528362	
rs2072862	IL2RB	22	37528606	x
rs228963	IL2RB	22	37535948	
rs228966	IL2RB	22	37537514	x
rs3218264	IL2RB	22	37541998	x
rs3218258	IL2RB	22	37544245	x
